# Production, Recycling and Economy of Palladium: A Critical Review

**DOI:** 10.3390/ma17010045

**Published:** 2023-12-21

**Authors:** Tomasz Michałek, Volker Hessel, Marek Wojnicki

**Affiliations:** 1 Faculty of Non-Ferrous Metals, AGH University of Krakow, Mickiewicza Ave. 30, 30-059 Krakow, Poland; tomaszm@agh.edu.pl; 2 School of Engineering, University of Warwick, Coventry CV4 7AL, UK; volker.hessel@adelaide.edu.au; 3 School of Chemical Engineering, The University of Adelaide, Adelaide 5005, Australia

**Keywords:** palladium, supply chain, production methods, recycling techniques, economic dynamics, emission regulations

## Abstract

Platinum group metals (PGMs), including palladium, play a pivotal role in various industries due to their unique properties. Palladium is frequently employed in technologies aimed at environmental preservation, such as catalytic converters that reduce harmful emissions from vehicles, and in the production of clean energy, notably in the hydrogen evolution process. Regrettably, the production of this vital metal for our environment is predominantly centered in two countries—Russia and South Africa. This centralization has led to palladium being classified as a critical raw material, emphasizing the importance of establishing a secure and sustainable supply chain, as well as employing the most efficient methods for processing materials containing palladium. This review explores techniques for palladium production from primary sources and innovative recycling methods, providing insights into current technologies and emerging approaches. Furthermore, it investigates the economic aspects of palladium production, including price fluctuations influenced by emission regulations and electric vehicle sales, and establishes connections between palladium prices, imports from major producers, as well as copper and nickel prices, considering their often co-occurrence in ores.

## 1. Introduction

**Figure 1:**
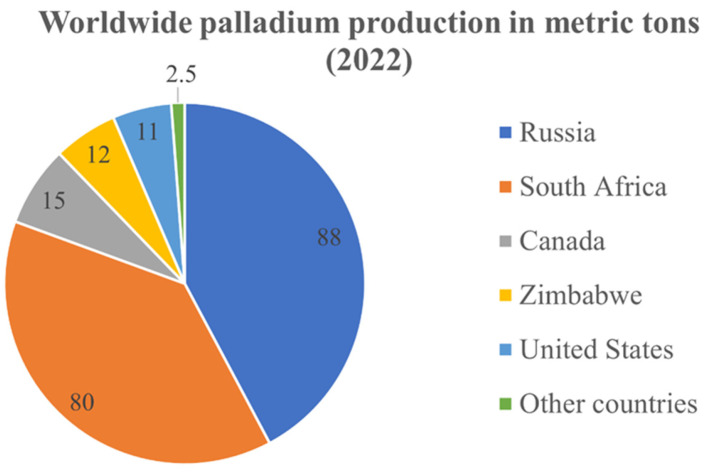
Palladium production by country in 2022, based on [10].

Platinum group metals (PGMs), a family of rare and valuable elements, play a pivotal role in a multitude of industries due to their exceptional properties. The collective demand for these metals is steadily escalating, driven by their indispensable contribution to modern technologies and emerging sustainable solutions. Among the PGMs, palladium stands as a prominent member, revered for its diverse range of applications that span across electronics [1], pharmaceuticals [2], and jewelry [3]. However, its most notable applications lie in its role in environmental preservation, such as catalytic converters that mitigate harmful emissions in the automotive sector [4] and as a catalyst for renewable energy production [5]. The extensive release of greenhouse gases leads to global warming, posing a significant challenge for environmental sustainability, a major concern for mankind today [6]. The imperative for a transition to green energy highlights the growing interest in clean, renewable energy from both industrial and academic standpoints [7]. Palladium and its alloys are recognized for their excellence in hydrogen production membranes, owing to their superior selectivity and permeation rate in comparison to other inorganic metallic membranes [8]. The role of palladium in preserving the environment is not limited to hydrogen production, as the researchers have succeeded in developing a Pd-catalyzed reaction for the efficient synthesis of fine chemicals under environmentally friendly conditions [9]. As industries continue to harness the unique attributes of palladium, the imperative to ensure its sustainable supply and utilization becomes increasingly pressing. However, securing a steady supply is not a simple task, as worldwide palladium production is nearly monopolized by Russia and South Africa [10]. Other countries that have a major contribution to the worldwide palladium production are Canada, Zimbabwe, and the United States. Detailed information about the amount of palladium produced in 2022 is shown in Figure 1.

The vulnerable supply chain led to classification of palladium as a critical raw material [11]. As it is not possible to become completely independent of palladium’s main producers, optimalization of production and recycling processes is vital to creating a stable and secure palladium availability. That is why we believe it is important to provide a detailed explanation of the technologies currently in use and to introduce promising novel approaches. Additionally, the availability of palladium is dependent on its price, which is why we decided to examine the economic aspects of palladium, highlighting its price dependence on factors such as emission regulations and electric car sales. We will also analyze the impact of the amount of palladium exported by the main producers on its price. Furthermore, we will establish a correlation between its price and the quantities imported from major producers, as well as a correlation between palladium’s prices and that of copper and nickel, considering their common co-occurrence in ores.

## 2. Production of Palladium from Primary Sources

**Figure 2:**
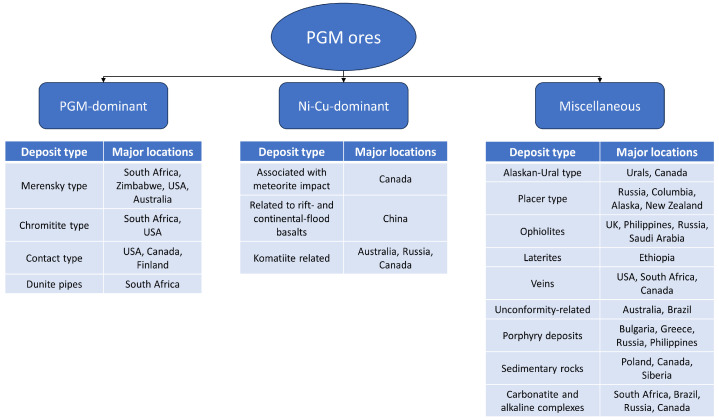
PGMs ore deposits’ classifications, based on [22].

Palladium ores often consist of complex mineral assemblages, in which palladium is typically found alongside other metals, such as nickel and copper [12], as well as other precious metals, including platinum, rhodium, and gold [13]. The mineralogy of these ores can vary widely, ranging from primary sulfide deposits to complex layered intrusions [14,15,16]. Palladium’s occurrence in these ores can take on various forms, including discrete mineral phases [17] or as part of solid solutions within host minerals [18]. Notable palladium-containing minerals include cooperite, braggite, and vysotskite [19]. The content of palladium in ores varies widely depending on the geological source and mineral composition; however, those ores can be classified into one of three groups: PGM-dominant ores, Ni-Cu-dominant ores, and miscellaneous ores [20]. Most of the PGMs’ global production originates from orthomagmatic sulfide deposits [21], which fall into the category of either PGM-dominant ores or Ni-Cu-dominant ores. In case of the former group, PGMs serve as the primary focus of extraction and are the main product of ore processing, whereas in the latter group, PGMs are produced as a valuable by-product alongside the primary extraction of nickel and copper [22]. That is why palladium production is closely related to nickel and copper production, which means that the demand for Ni and Cu will impact the price of PGMs [23]. Different types of ore deposits, and their classification in the mentioned groups, are presented in Figure 2.

Several approaches may be used for palladium production. Hydrometallurgy represents a metallurgical approach involving the separation and extraction of metals through reactions in water-based solutions [24]. Processes such as leaching, ion exchange, solvent extraction, and precipitation fall under the purview of hydrometallurgy. These methods present distinct advantages over the pyrometallurgical approach, including the capacity to process intricate ores, lower energy consumption, and diminished emissions of gaseous pollutants [25]. Furthermore, hydrometallurgy can assist in the processing of ores containing specific impurities that might pose challenges during smelting [26]. An additional significant consideration stems from the dwindling average metal content in ores, as reserves of higher-grade metal ores gradually deplete [27]. The hydrometallurgical approach is particularly adept at extracting metals from ores characterized by low metal concentrations, a task wherein pyrometallurgy may encounter obstacles. However, it is important to note that pyrometallurgy has some advantages over hydrometallurgy, mainly faster reaction rates due to elevated temperatures, which translates to high production rates. In hydrometallurgical methods, large amounts of chemicals are used, generating significant quantities of waste acids and sludge that must be carefully managed and disposed of as hazardous waste [28]. 

### 2.1. Obtaining Palladium from PGM- and Ni-Cu-Dominant Ores

In the case of those ores, depending on the PGMs’ concentration, palladium is either obtained as one of the main products or as a by-product during copper and nickel sulfide ores’ pyro/hydrometallurgical process. It will generally involve the following steps: beneficiation, smelting, conversion, and refining [29], although some preliminary steps are necessary, such as mining the ore and grinding it for easier processing.

#### 2.1.1. Beneficiation

Beneficiation is the process of improving the quality and value of an ore or raw material by various techniques to increase its concentration of valuable minerals or metals while reducing the content of unwanted impurities. In the case of PGM-containing ores, froth flotation is the most commonly used. It involves creating a froth on the surface of water to collect and separate hydrophobic mineral particles from hydrophilic gangue particles. Some chemical reagents are added, the most common being sulfhydryl-based collectors, alcohol-based frothers, and polymer-based depressants [30]. Air bubbles are then introduced into the system, and these hydrophobic particles become attached to the bubbles, rising to the froth layer on the surface. The froth, containing the valuable mineral particles, is skimmed off, while the gangue particles remain in the bulk solution. This process generally results in recovery of over 80% PGMs contained within the ore [31].

Recent innovations in the process of froth flotation are mainly focused on developing new collectors, with nanoparticles emerging as a very promising solution [30,32,33,34], offering exceptional recovery rates of certain metals, as well as high selectivity for them.

#### 2.1.2. Smelting

Smelting is a fundamental metallurgical process used to extract metals from their ores by heating the ore in a furnace. This process involves the application of high temperatures and often involves the addition of flux (e.g., SiO_2_) to facilitate the separation of the desired metal from other components in the ore [35], which in this case is mainly iron. A substantial amount of iron can be transferred to slag, and in the case of, Outokumpu flash furnace blister copper may be produced directly without the need of converting [36]. Major reactions that occur during smelting can be described by Equations (1) and (2):

(1) $$CuFeS_{2}+O_{2}+SiO_{2}\to [Cu-Fe-S{]}_{matte}+(FeO\cdot SiO_{2}{)}_{slag}+SO_{2}$$

(2) $$NiS+FeS+O_{2}+SiO_{2}\to [Ni-Fe-S{]}_{matte}+(FeO\cdot SiO_{2}{)}_{slag}+SO_{2}$$

It should be noted that palladium, along with other PGMs, will be collected within the matte, a mixture of metal sulfides, produced during the smelting of sulfide ores. The reason lies in the palladium distribution coefficient values between copper/nickel matte and a slag (*L*^[*Cu/Ni*]*/S*^). This coefficient represents the ratio of palladium extracted to the metallic phase against palladium contained within the slag after the smelting process [37], as presented in Equation (3):

(3) $$L^{[Cu/Ni]/S}(Pd)=\frac{[Pd\;wt\%{]}_{Cu/Ni}}{(Pd\;wt\%{)}_{S}}$$

Palladium must undergo oxidation to be extracted into the slag phase. The corresponding oxidation reaction has been presented in Equation (4):

(4) $$Pd+\frac{1}{2}O_{2}=PdO$$

When the oxide in the slag is presented as a monocation form (MO_x_), Equation (3) can be easily transformed into another form, incorporating oxygen partial pressure (*pO*_2_) [37]. This parameter is of utmost importance in determining the extent to which the metal will be oxidized and transferred to the slag phase [38]. For the reaction presented in Equation (4), the distribution coefficient can be expressed by the following Equation (5):

(5) $$L^{[Cu/Ni]/S}(Pd)=\frac{\left[n_{Total}](\gamma_{PdO}\right)}{K\left(n_{Total}\right)\left[\gamma_{Pd}\right]pO_{2}}$$

where *K* is the equilibrium constant for the reaction shown in reaction (4), [*n_Total_*] and (*n_Total_*) represent the total number of moles of species in the metal matte and slag phase, respectively, and [*γ_Pd_*] and (*γ_PdO_*) are the activity coefficients of palladium in the metal matte and PdO in the slag, respectively.

As the oxygen partial pressure increases, more palladium will be oxidized and, consequently, extracted into the slag phase. Generally, the smelting process is conducted at low values of *pO*_2_. This implies that the distribution coefficients for palladium production will be quite high, indicating that palladium will be extracted into the metallic phase. Some values of *L^[Cu/Ni]/S^* from the literature have been organized in Table 1.

*L^[Cu/Ni]/S^* values presented in Table 1 are remarkably consistent. The evident conclusion is that only a negligible amount of palladium is extracted into the slag phase. Therefore, the subsequent necessity for achieving pure palladium involves the refining of copper/nickel.

#### 2.1.3. Converting

Metal matte undergoes a conversion process, as it has high concentrations of iron and sulfur, which have to be removed before the refining [43]. This is carried out in reactors called converters, in which air is blown through molten metal matte and flux [44]. This process results in oxidation of iron and sulfur, which are subsequently removed from the matte. There are many types of converters that can be used in this process, such as the Peirce–Smith converter [44], bottom blowing converter [45], Kennecott–Outotec flash converter [46], and the Ausmelt converter [45]. The Peirce–Smith converter and its modifications are the most common due to their simplicity of operation [44]. To an extent, PGM losses during this process are unavoidable, especially if the processed matte is of low quality. When the matte contains more iron to be removed into slag, some PGMs may also be moved there due to the sheer effect of the law of mass action [47]. Final products of the conversion are either high-grade metal matte or blister metal.

#### 2.1.4. Refining

The final step, in which the palladium can finally be obtained, is leaching (in case of nickel) or electrorefining (in case of copper). Note that the ultimate goal for those processes is usually to obtain pure copper/nickel, while palladium is simply recovered (along with other PGMs). Leaching is a process of extracting valuable metals from solid phase using liquid solvents [48]. Due to the unique properties of PGMs, mainly their high chemical resistance, they can be separated from nickel without much effort, usually by leaching nickel while leaving PGMs intact in the solid residue [49]. In the case of copper production, palladium is recovered during the electrorefining process. It consists of electrochemically dissolving blister copper (used as an anode) in a mixture of H_2_SO_4_ and CuSO_4_, and then subsequently depositing cathodic copper on stainless steel [50]. Palladium is contained within anodic slime, among other valuable metals [51]. Then, it is only a matter of separating palladium from other metals, which can be conducted via solvent extraction or selective precipitation (after dissolving them in chloride-based solutions) [52]. 

### 2.2. Obtaining Palladium from Miscellaneous Ores

In the case of miscellaneous ores, we are referring to those containing PGMs in exceptionally low concentrations. Therefore, palladium is not recovered from them, or if recovered, it is obtained as a by-product but with little economic gain [53]. Developing techniques for palladium recovery from those ores is a topic of interest among metallurgists, as collectively they contain a sizeable amount of PGMs, which could potentially be extracted in the future. However, it is not an easy endeavor, as those ores differ in mineral compositions, the form of PGMs contained within them, and the types of gangue present. Even if a method of extracting palladium from one type of ore would be developed, it is highly unlikely that it would work sufficiently for other ores in this group.

### 2.3. Obtaining Palladium from Oxidized Ores

Oxidized ores are not technically distinct as a separate category of PGM ores, since they typically form as a part of near-surface deposits within other ore groups. Consequently, they have undergone weathering and oxidation processes, leading to the creation of PGM oxides and hydroxides, along with associated metals [54]. The production of platinum group metals from oxidized ores is a formidable undertaking, since conventional techniques are inadequate in terms of efficiency for achieving cost-effective production [55]. The reasons are the highly heterogeneous and polymodal distribution of PGMs and the absence of associations with base metal sulfides. Another reason is the presence of naturally floating gangue in those ores, which drastically decreases the resulting concentration of PGMs in the produced concentrates, and the usage of conventional gauge depressants decreases PGMs’ recovery [56]. Thus, the hydrometallurgical route seems to be the optimal solution for treatment of those ores. There is no standardized method for extracting PGMs from these ores, considering their variations in both phase and elemental composition, which may be the case even for ores from a single deposit [57].

Kraemer et al. [58] proposed a two-step leaching procedure to recover platinum and palladium from oxidized PGM ores. The initial step entails a hydrochloric acid pretreatment to release the palladium-bearing host phases from weathering by-products and decrease the presence of easily leached metals, such as iron, which might contend with palladium for binding to DFOB during the subsequent stage. The second stage involves a siderophore leaching process utilizing the biogenic ligand Desferrioxamine B (DFOB) to extract platinum and palladium from the ore. The study’s findings indicate potential palladium recoveries of up to 25%, and up to 80% for platinum. 

Mpinga et al. [59] proposed a two-stage procedure as well, although in their case it was a hybrid pyro–hydro process. It begins with a sulfation roast or a salt chlorination roast pretreatment, followed by an acid chloride leach of the oxidized ore. The focus was primarily on platinum, given its reputation for being more resistant to oxidation and dissolution compared to other precious metals; however, they managed to extract an impressive amount of palladium (93.3%), while simultaneously extracting a very negligible amount of iron (16.9%). They also proposed a single-stage salt chlorination process, in which they achieved complete leaching of palladium (100%) within 30 min, without the addition of further oxidizing agents. Other benefits of this single-stage process are simultaneous complete extraction of platinum within 3 h, as well as extracting high amounts of nickel and copper, and only about 30% of iron after 24 h.

The bioleaching pretreatment process proposed by Hedrich et al. [54] involves the utilization of microbial communities with the addition of elemental sulfur to support their growth. This resulted in nearly complete extraction of base metals, leaving most of the PGMs in the solid phase. The obtained residue was subsequently subjected to HNO_3_/NaCl leaching, leading to the achievement of a maximum extraction of 89% platinum and 96% palladium under optimal pretreatment conditions. Another residue-treatment process they conducted was cyanide leaching, which also demonstrated an impressive extraction of 81% platinum and 76% palladium. These figures surpass the extraction results from attempts conducted without bioleaching pretreatment, where HNO_3_/NaCl as well as cyanide leaching resulted in extraction rates of about 44% for platinum and 56% for palladium.

An alternative strategy for dealing with oxidized ores involves their pretreatment. In this method, instead of leaching the ores after pretreatment, they undergo a flotation process, resulting in the production of a concentrate. Numerous literature sources highlight successful elimination of certain base metal oxides from oxidized ores, employing techniques such as acid pretreatment [60,61], sulfidization [62,63], and cyanide leaching [64,65]. Nonetheless, the concept of pretreating these ores for the purpose of flotation is currently an area with a limited research focus.

## 3. Recycling of Palladium

**Figure 3:**
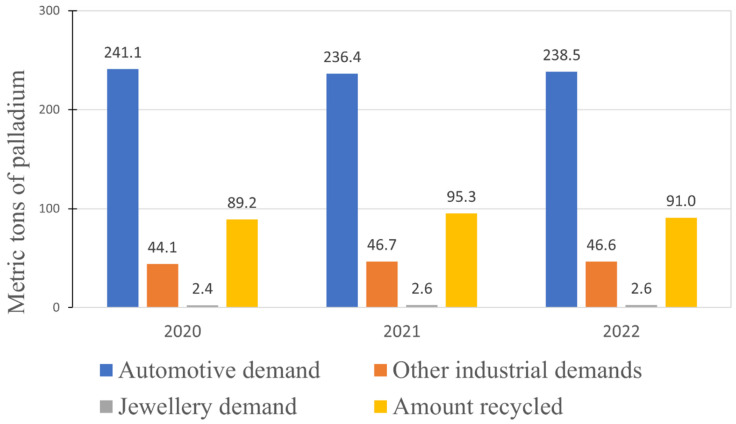
Palladium demand in various sectors in recent years, as well as the amount of palladium recycled, based on [67].

The recovery of PGMs from primary ores faces challenges, such as low PGMs content, high energy consumption, and limited concentration recovery. Conversely, secondary resources boast significantly higher PGM concentrations, which makes them highly preferable for exploitation, while simultaneously curbing the need for extensive ore mining. Secondary sources demand significantly less energy consumption, attain superior recovery yields, and bear a diminished environmental footprint [66]. It might be assumed that the recycled materials for palladium recovery would exhibit significant variability. However, there is one main type of material that stands out. To explain this, we present data regarding the palladium demand across various sectors, and how much palladium was recycled from 2020 to 2022 (shown in Figure 3).

As shown in Figure 3, the greatest demand for palladium lies in the automotive sector, which is why the main resources for palladium recycling are spent automotive catalytic converters. These converters contain significant amounts of precious metals, including palladium, which are used as catalysts to reduce harmful emissions from vehicle exhaust gases. Since spent catalytic converters are discarded once they are no longer effective, they become a valuable secondary source of palladium. Other notable applications of palladium are in the chemical and electric industries [68]. An additional observation to be made from Figure 3 is that the presented data are quite constant over the presented time span. Shares of palladium demands in individual sectors stay relatively the same, which is also true for the amount of palladium recycled, indicating a lack of significant breakthroughs in the recovery methods. For years, the pyro/hydrometallurgical process has been dominating PGMs’ recycling [69] (similar to their production from ores depicted in Section 2.1), although purely hydrometallurgical methods are constantly researched.

### 3.1. Pyro/Hydrometallurgical Process

The process of recovering palladium through pyro/hydrometallurgical methods is comparable to that outlined for sulfide ores in the preceding section (Section 2.1). This approach is widely used for treating spent automotive catalysts [70]. The fundamental principle involves smelting the ground material with the addition of flux. However, due to the differing composition compared to ore concentrates, metal collectors are introduced to aid in the extraction of palladium into the metallic phase [71]. This results in the creation of a PGM-enriched alloy [72]. Subsequent refining steps enable the production of metallic palladium.

Volatilization processes present an alternative to traditional smelting, in which PGMs are volatilized by selective chlorination and condensed in a cooler zone [72]. While this process provides high selectivity by exploiting the differences in vapor pressures of metal chlorides, it also leads to severe furnace corrosion. Another downside of this process is the presence of CO and Cl_2_ gases, which pose environmental risks.

Another notable example of pyrometallurgical operation used to recover palladium from spent catalysts is plasma sintering. It is a process that involves the use of a plasma furnace and plasma-torch assembly to sinter material, leading to in situ reduction of its oxidized PGM components [73]. This method is great for reforming spent catalysts but is not suitable for industrial-scale production of palladium.

### 3.2. Hydrometallurgical Process

The hydrometallurgical recycling of spent automotive catalysts is presently a vigorously researched topic, as indicated by the SciVal tool (https://www.scival.com/trends/summary?uri=Topic/14964) provided by Elsevier. During the period from 2018 to 2022, Topic T.14964 (Catalyst; Hydrochloric Acid; Catalytic Converters), which falls under Topic Cluster TC.656 (Solvent Extraction; Leaching; Liquid Membranes), achieved a Scholarly Output of 341 publications and a Prominence Percentile of 94.313 [74]. The reasons for the substantial interest in catalyst recycling through the hydrometallurgical route are twofold: the achievement of high recovery rates of PGMs [75] and the potential for producing Pt/Pd-rich solutions that can serve as valuable end products [76]. These solutions find application as precursors in the synthesis of various materials, primarily other catalysts, thereby eliminating the need to reduce these elements into metals during recycling.

Following the initial preparations, primarily milling, the first step in recycling spent catalysts involves leaching. Considering that platinum and palladium fall under the category of noble metals, their leaching in HCl poses difficulties. This process hinges on the modification of the oxidation potential, commonly by prior roasting [77], or by leaching in the presence of H_2_O_2_, which enables the creation of stable aqua chloro-complexes, thus facilitating their dissolution [78]. In the case of palladium, the following reaction, shown in Equation (6), occurs:

(6) $$Pd+H_{2}O_{2}+4HCl\to [PdCl_{4}{]}^{2-}+2H^{+}+2H_{2}O$$

It is important to note that the dominance of various forms of palladium complexes can vary depending on the concentration of chloride ions. In some cases, these complexes may replace H_2_O as ligands instead of chloride ions [79].

The leaching process of palladium from spent automotive catalysts is a non-catalytic, heterogeneous solid/liquid reaction. The reaction rate, *v*, can be expressed as the moles reacted or formed per unit surface area over time [80,81], as presented in Equation (7):

(7) $$v=-\frac{1}{bA_{Pd}}\left(\frac{dN_{Pd}}{dt}\right)$$

where *b* is a stoichiometric coefficient, *A_Pd_* is the reacting area of the palladium grain surface, *N_Pd_* is the number of moles of the solid palladium phase, and *t* is the time.

By reformulating Equation (7) using the conversion *X*, which is a measure of the extent to which a reaction has occurred (*N_Pd_ = N_Pd0_* (1 − *X*)), we obtain the expression given in Equation (8):

(8) $$v=\frac{N_{Pd0}}{bA_{Pd}}\left(\frac{dX}{dt}\right)=\frac{\rho_{Pd}V_{Pd0}}{bA_{Pd}}\left(\frac{dX}{dt}\right)$$

where *ρ_Pd_* is the molar density of palladium and *V_Pd_*_0_ is the initial volume of the solid palladium particles.

The reaction rate is further influenced by the concentrations, *C*, of the reactants. This relationship is governed by the rate constant (*k*), in accordance with the law of mass action [82], as expressed in Equation (9):

(9) $$v=kC_{Pd}^{n}C_{A1}^{a1}C_{A2}^{a2}$$

where *A*1 and *A*2 are the leaching reagents (e.g., HCl and H_2_O_2_, as presented in reaction (3)), and parameters *n*, *a*1, and *a*2 are the reaction orders specific to each reagent. As palladium is present in a solid state, *C_Pd_* does not refer to concentration in a solution but rather to the moles of palladium per unit volume in a solid.

If a safe excess of leaching reagents is present in a system, and we assume that their concentrations remain constant throughout the reaction, then only solid palladium particles will be expressed as a function of the conversion (*C_Pd_ = C_Pd0_* (1 − *X*)). With this assumption, we can combine reactions (5) and (6), resulting in Equation (10):

(10) $$\left(\frac{dX}{dt}\right)=\frac{bA_{Pd}C_{Pd0}^{n}C_{A1}^{a1}C_{A2}^{a2}}{\rho_{Pd}V_{Pd0}}k(1-X{)}^{n}=k_{R}(1-X{)}^{n}$$

where *k_R_* is an apparent rate constant that encompasses all parameters remaining constant over time.

The final step involves integrating Equation (10), resulting in a conclusive model that can be employed to describe experimental data obtained during palladium leaching, as expressed in Equation (11):

(11) $$\frac{1}{n-1}[\left(1-X{)}^{1-n}-1\right]=k_{R}t,\;\;\;\;n\neq 1$$

It is important to note that other, more general models may also be applicable to the process of leaching palladium, such as the Shrinking Core Model [83,84] or the Shrinking Particle Model [85]. However, both of these models assume that solid particles have a predetermined, uniform geometry, which is not always the case.

Several noteworthy novel methods for leaching palladium from spent catalysts have emerged, including ultrasonic-enhanced ozonation in HCl [83], leaching using ionic liquids [86], and microwave-assisted leaching [87], all of which resulted in remarkably high palladium leaching rates. Bioleaching enables recovery of PGMs as well; however, due to poor efficiency, it is not applicable to wide-scale industrial operations [86].

The next step after the leaching process typically involves solvent extraction, which allows for the separation of palladium from other metals using ionic liquids [87]. A solvent extraction setup typically comprises two phases that do not mix: an organic phase, which holds the extractant, diluent, and potentially a modifier, and an aqueous phase, which contains the metals targeted for separation. Considerable research effort is being directed toward enhancing metal separation in a range of strategies, such as the formulation of novel extractants, the utilization of extractant blends, modification of diluents, incorporation of modifiers, and more [88]. Metal ions contained within organic phase can then be extracted back to aqueous phase by stripping [89].

Finally, the last step is producing metallic palladium, which is a relatively easy task. It can be achieved by variety of methods, such as precipitation [77], cementation [90], adsorption [70], and others.

## 4. Economy of Palladium

The economic landscape surrounding palladium is shaped by a complex interplay of factors that dictate its market dynamics and value. As a sought-after precious metal, palladium’s economic significance is underscored by various elements that range from regulatory policies to intricate market correlations.

The palladium market is notably influenced by environmental regulations and emission legislation. These regulations, aimed at curbing harmful pollutants from various sources, particularly vehicles, have a direct impact on palladium demand. As the automotive industry adapts to stringent emission standards, the requirement for palladium-intensive catalytic converters surges. On the other hand, electric car sales are steadily increasing, and since they do not require catalytic converters, this trend is expected to decrease demand for palladium.

The correlation between palladium supply and its price is another crucial aspect of its economy. Supply disruptions, often stemming from factors such as mining strikes, geopolitical uncertainties, and labor disputes, can significantly affect the global palladium market. These disruptions can lead to supply shortages, propelling palladium prices upward due to the constrained availability of the metal.

Moreover, palladium’s relationship with other commodities, such as copper, adds complexity to its economic dynamics. Palladium and copper share certain market characteristics, and their prices can exhibit correlated movements. Understanding this correlation provides valuable insights into how palladium prices might react to changes in the broader commodities market.

### 4.1. Influence of Emission Legislation

Emission legislation refers to laws, regulations, and standards put in place by governments and international organizations to control and limit the amount of pollutants and harmful substances released into the environment, particularly from various industrial activities, vehicles, and other sources. These legislations are designed to address environmental concerns, protect public health, and mitigate the negative impacts of pollution on ecosystems and human well-being [91,92]. The connection between emission regulations related to vehicle emissions and the demand for palladium is strongly tied to palladium’s crucial role as a key component in catalytic converters [93]. These converters play a vital part in reducing harmful emissions from vehicles, and so the growing stringency of emission legislation should require an increased palladium presence in catalytic converters.

Emission Standards in the European Union, published by the European Commission, were implemented in stages (applied to new passenger car approvals) [94,95]:

- Euro 1—Approved in July 1992, first registration in January 1993 (although technically in September 1992).
- Euro 2—Approved in January 1996, first registration in January 1997.
- Euro 3—Approved in January 2000, first registration in January 2001.
- Euro 4—Approved in January 2005, first registration in January 2006.
- Euro 5—Approved in September 2011, first registration in January 2013.
- Euro 6—Approved in September 2014, first registration in September 2015.

Commencing from 2017, the European Union incorporated novel Real-Driving Emissions (RDE) tests into its emission-type approval process for passenger cars, utilizing onboard portable emissions measurement systems (PEMS) [96]. While not being a legislation, incorporation of those tests should theoretically increase the demand for palladium as well.

Emission Standards in the United States, published by the U.S. Environmental Protection Agency, were implemented as tiers [97,98]:

- Tier 1—Published in June 1991, start of implementation in the year 1994.
- Tier 2—Published in December 1999, start of implementation in the year 2004.
- Tier 3—Published in March 2014, start of implementation in the year 2017.

**Figure 4:**
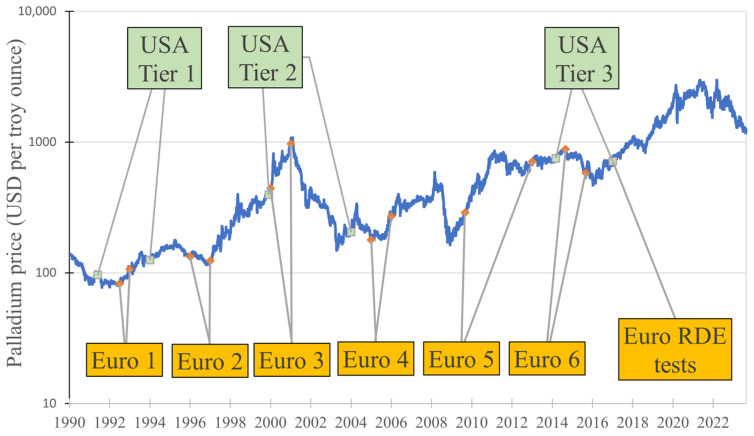
Changes in the price of palladium in the context of emission legislation.

The influence of implementing the mentioned legislations (as well as the EU RDE test) on palladium prices is visualized in Figure 4 (price data obtained from Macrotrends [99]).

Analyzing the course of the graph presented in Figure 4 along with the plotted points associated with the introduction of emission legislation, the following conclusions can be drawn:

- The announcement of USA Tier 1 caused a temporary spike in palladium prices; after its implementation, the price steadily grew.
- Both the approval and first registration of Euro 1 resulted in an increase in palladium prices.
- The announcements of Euro 3 and USA Tier 2 (with a slight time deviation from each other) were followed by a significant rise in the price of palladium, which peaked at 1075 USD just days after the first registration of Euro 3. Subsequently, prices declined; however, after the implementation of USA Tier 2, another price peak occurred.
- The approval of Euro 4 had no immediate effect on palladium prices. However, prior to its first registration and a few years after, prices began to steadily rise.
- Between 2008 and 2009, palladium prices drastically decreased, but in 2009, they began to steadily increase. This increasing trend continued after the approval of Euro 5, peaking in late July 2021 at 836 USD per troy ounce, and then began to slightly decline. The first registration of this legislation had no significant effect on palladium prices.
- After the announcement of USA Tier 3, the price of palladium began to rise slightly, peaking at the approval date of Euro 6. The first registration of Euro 6 resulted in a momentary increase in the price of palladium, followed by a decline for a short time, and then a steady increase. Throughout this increase, both USA Tier 3 and Euro RDE tests were implemented, both of which likely contributed to sustaining the price increase.

### 4.2. Influence of Electric Cars Sales

**Figure 5:**
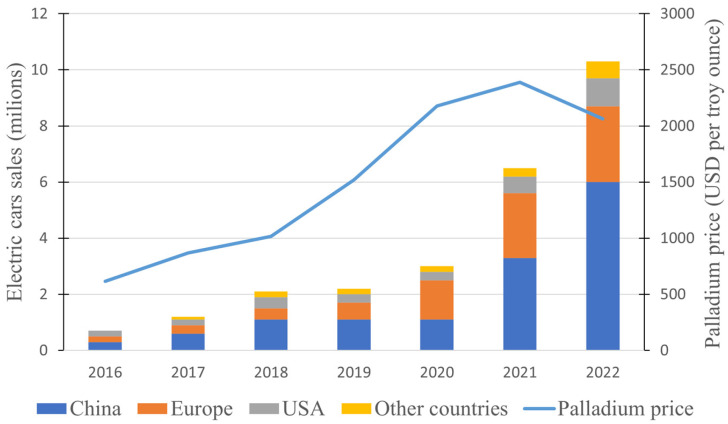
Yearly sales of electric cars and yearly average prices of palladium spanning from 2016 to 2022.

Electric cars, also known as electric vehicles (EVs), are a revolutionary form of transportation powered primarily by electricity instead of traditional internal combustion engines that rely on gasoline or diesel [100]. They utilize advanced battery technology to store and deliver electricity to an electric motor, which propels the vehicle [101]. This shift to electric propulsion brings several benefits, including significantly lower emissions compared to conventional vehicles, reduced reliance on fossil fuels, and quieter and smoother operation [102]. The surge in electric car adoption is driven by factors such as environmental concerns, government incentives, advancements in battery technology, and the growing demand for sustainable transportation options. The ascent of electric vehicles presents an intriguing avenue for exploration regarding their potential to influence palladium prices. Unlike conventional internal combustion engine vehicles, electric cars do not require catalytic converters, a key component that accounts for a significant portion of palladium demand. As the popularity of electric vehicles grows, the diminished reliance on palladium-containing catalytic converters could potentially result in a decline in palladium demand, thus introducing a noteworthy variable to its pricing dynamics. In Figure 5, we present annual numbers for electric cars sold alongside the yearly average palladium price, aiming to visualize a potential influence.

At first glance, it appears that the rising sales of electric cars have had no discernible impact on palladium prices. While the number of EV sales increased gradually from 2016 to 2020, the palladium price also rose. An interesting shift occurred since 2021, with a substantial surge in EV sales; while the palladium price continued its upward trajectory, the rate of increase showed a slight easing. In 2022, marked by another noteworthy surge in sales, a decline in palladium prices was noted, and this downward trend persists as of June 2023. While these data do not explicitly demonstrate that this price drop was mostly a result of a decrease in palladium demand for the automotive industry, it is quite possible that it is a major contributing factor.

### 4.3. Correlation between Palladium Supply and Its Price

**Figure 6:**
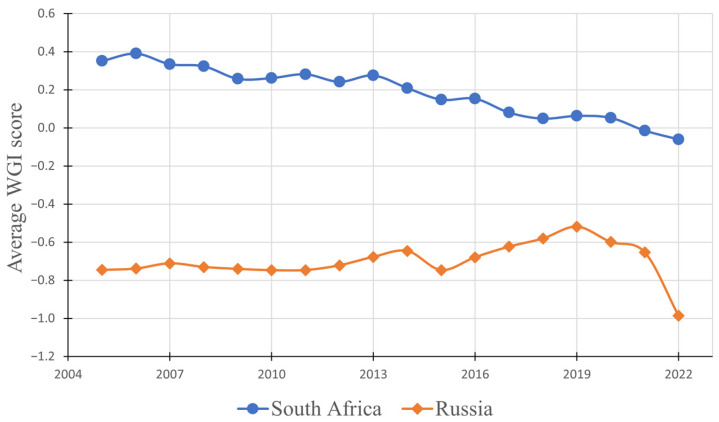
Yearly average WGI scores of South Africa and Russia, spanning from 2005 to 2022, based on [106].

At the core of commodity markets lie fundamental principles of supply and demand, dictating price fluctuations and market behavior. In this section, we delve into a critical aspect of these dynamics: the intricate relationship between palladium prices and the exported quantities from Russia and South Africa. These two major producers wield substantial control over palladium supply, making their contributions crucial in shaping market trends. Unfortunately, this means that the palladium supply chain is vulnerable. If either of these countries were to limit their palladium production and exports, it would drastically reduce the global supply. Currently, the most pressing risk in the global mining industry is the restriction of Environmental, Social, and Governance (ESG) practices [103]. The likelihood of supply interruptions can be indicated by the supplier’s ESG performance, which can be quantified by the World Governance Index (WGI) and Environmental Performance Index (EPI) [104]. South Africa’s EPI score in 2022 was 37.2, resulting in a ranking of 116 among the 180 countries evaluated, while Russia achieved an EPI score of 37.5 with a ranking of 112 [105]. Scoring this low corresponds to high environmental risk, implying a high probability of negative environmental impacts in the supply process. Concerning the WGI, the score is on a scale of −2.5 (weak governance performance) to 2.5 (strong governance performance). The WGI scores of South Africa and Russia have been organized in Figure 6.

Figure 6 shows that South Africa’s WGI score has steadily declined since 2005, reaching a negative value in the year 2021. Russia’s WGI score is lower than that of South Africa and has sharply declined to a value of −0.97 in the year 2022. This is likely a result of Russia’s war with Ukraine. An interesting thing to note is that Russia’s WGI score declined in all of the evaluated categories compared to 2021, with the lowest scores being in Voice and Accountability (−1.26) and Rule of Law (−1.20). Russia’s government undeniably poses a higher risk to the global palladium supply chain than that of South Africa. However, as South Africa’s WGI score has reached a subzero value, and the overall trend suggests that it may continue to decline, it can be said that the overall palladium supply chain is vulnerable.

Our analysis centers on the perspective of palladium imports to the United States, drawing insights from data provided by the United States Geological Survey [10]. By exploring this correlation, we aim to unravel potential connections between exports from these nations and the pricing landscape of palladium, providing a nuanced understanding of the underlying forces driving the market. Utilizing global data would yield greater precision; nevertheless, obtaining such data proves challenging, and the US market’s substantial size renders it capable of providing a representative sample.

**Figure 7:**
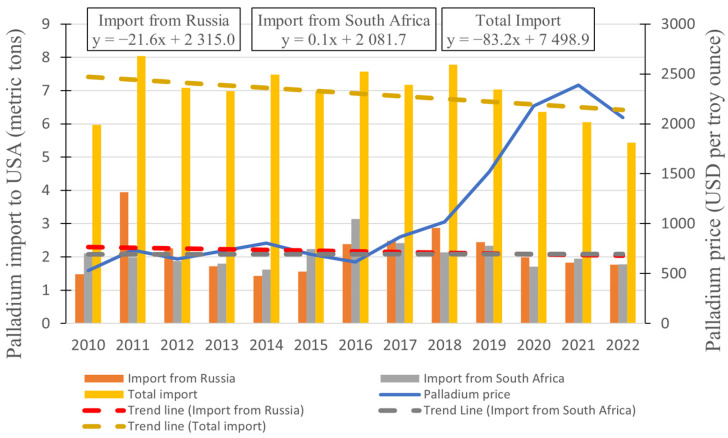
Yearly average values of palladium imports to the USA and yearly average prices of palladium spanning from 2010 to 2022.

In order to explore the possible interdependence between palladium imports and price dynamics, we examined the yearly average quantities of palladium imported into the United States from 2010 to 2022. Simultaneously, we considered the average annual prices of palladium over the same timeframe. These two sets of data are presented in Figure 7, allowing us to visually grasp potential correlations and trends between import volumes and price fluctuations.

During the time span depicted in Figure 7, it becomes evident that the overall import of palladium to the USA is declining, while the quantities imported from Russia and South Africa remain relatively constant. Nevertheless, the decline in palladium prices from 2021 to 2022 cannot be attributed to a sudden surge in supply, given that the imports during those years were, in fact, smaller than in the preceding years. To determine the precise correlations, we used the Pearson model. Correlation coefficient values (presented in Table 2) were calculated using Equation (12):

(12) $$r_{xy}=\frac{n\sum x_{i}y_{i}-\sum x_{i}\sum y_{i}}{\sqrt{n\sum x_{i}^{2}-(\sum x_{i}{)}^{2}}\sqrt{n\sum y_{i}^{2}-(\sum y_{i}{)}^{2}}}$$

where *n* is sample size and *x_i_* and *y_i_* are the individual sample points, indexed with “*i*”.

The values presented in Table 2 reveal intriguing interdependencies. Notably, it appears that imports from Russia exert negligible influence on palladium prices, with a value of −0.15 signifying a very weak negative correlation (where a value of 0 indicates no correlation and 1 implies a perfect correlation). This suggests that an increase in palladium imports from Russia would lead to a minimal decline in palladium prices. A similar scenario emerges with South Africa, where a value of −0.34 also indicates a small correlation, albeit slightly more noticeable than that with Russia. However, a distinct pattern emerged when considering total imports. Evidently, a robust correlation exists, with a value of −0.61, surpassing the 0.5 threshold that denotes a moderate correlation. From this data, one can infer that the USA has effectively diversified its sources of palladium, exhibiting resilience against short-term supply disruptions from the primary palladium producers. In summary, the analysis suggests that the USA has adeptly managed to diversify its palladium sources, thereby displaying remarkable resilience in the face of potential supply challenges from key palladium producers.

### 4.4. Correlations between the Prices of Copper and Nickel, and the Price of Palladium

**Figure 8:**
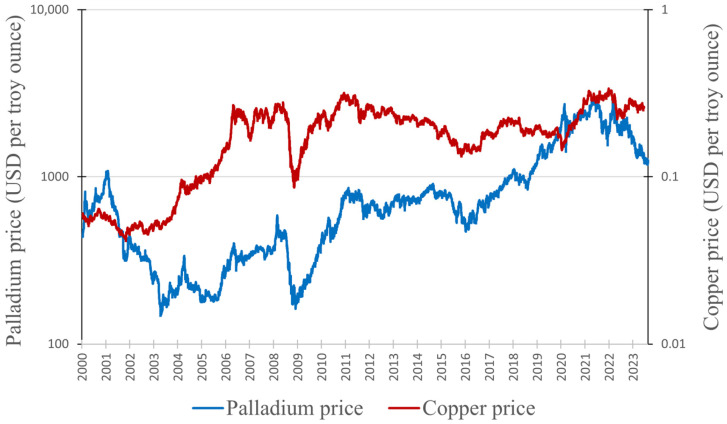
Changes in the prices of palladium and copper.

**Figure 9:**
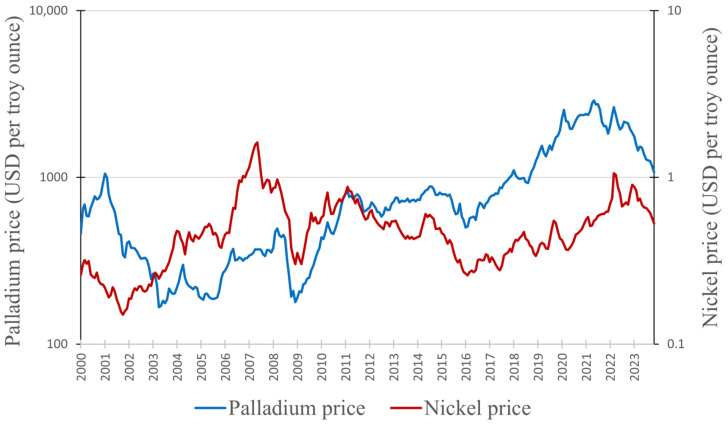
Changes in the prices of palladium and nickel.

In this section, we will examine the connection between the prices of copper and nickel and the price of palladium. As mentioned earlier (Section 2.1), copper and nickel are frequently found in the same ores as palladium, resulting in an interconnected supply. This interdependence is the reason for a potential correlation between their prices. Figure 8 presents a graph depicting the prices of copper and palladium from January 2020 to August 2023 (data obtained from Macrotrends [99,107]). Figure 9 presents the prices of nickel and palladium (data for nickel obtained from [108]). Unfortunately, we were not able to obtain as precise data for nickel as we did for copper and palladium; hence, prices are presented as monthly averages.

Clear correlations between the prices of palladium and copper are evident in Figure 8. A notable instance is the significant price drop of both metals in 2009, followed by a steady growth that peaked in 2011. Another example is the price drop in 2016, succeeded by a steady increase until 2018. To determine the precise correlation, we once again utilized the Pearson correlation coefficient in the form of Equation (4). The correlation of copper prices (*x*) with palladium prices (*y*) resulted in *r_xy_* = 0.51. The value of nearly exactly 0.5 indicates a moderately positive linear relationship, which means that the increase in the copper price had a clear impact on palladium prices.

Figure 9 illustrates that there are indeed some analogies when comparing the prices of palladium and nickel. The main similarity is the occurrence of price drops in 2009 and 2016, akin to the case of copper. Another parallel in the price history of nickel and palladium is a steady decline after the year 2022. However, there are many differences, such as the intense price peak of nickel in 2007 (which was actually its highest price during the analyzed period). Such a peak in that year cannot be found for palladium. The Pearson correlation value for the prices of nickel (*x*) and palladium (*y*) that we obtained was *r_xy_* = 0.12. Even though the data used here are less precise than in the case of copper and palladium (since we were operating on monthly average prices), this still indicates a very weak positive linear relationship between these variables. Changes in the price of nickel will have a minimal impact on the price of palladium.

## 5. Conclusions

Recent research trends highlight a growing interest in hydrometallurgical recycling methods, aiming for high recovery rates of precious group metals and the production of valuable Pt/Pd-rich solutions for various applications. Palladium-containing ores encompass a wide spectrum of mineral compositions, including PGM-dominant, Ni-Cu-dominant, miscellaneous, and oxidized ores, reflecting the diverse geological sources of this precious metal. Extraction from PGM- and Ni-Cu-dominant ores involves beneficiation, smelting, converting, and refining, with palladium primarily recovered as a by-product. Miscellaneous ores pose economic challenges due to low PGM concentrations, requiring ongoing research for efficient extraction techniques. Oxidized ores demand specialized approaches, with hydrometallurgy showing potential for effective palladium recovery.

Recycling of palladium offers a sustainable and circular alternative to primary ore extraction, with secondary resources providing higher PGM concentrations and reduced environmental impact. The primary source of recycled palladium is spent automotive catalytic converters, reflecting the substantial demand for palladium in the automotive sector. These converters contain significant amounts of precious metals, including palladium, which serve as catalysts to reduce harmful emissions from vehicle exhaust gases. The recycling process encompasses both pyro/hydrometallurgical and hydrometallurgical methods, each with its unique approach to recovering palladium from secondary resources. Pyrometallurgy involves smelting and alternative methods, such as volatilization processes and plasma sintering. Hydrometallurgy includes leaching, solvent extraction, and the final step of producing metallic palladium, often achieved through precipitation or cementation.

Environmental regulations, particularly in the automotive industry, drive demand for palladium-intensive catalytic converters, shaping palladium’s economic landscape. The rise of electric vehicles presents a potential challenge to palladium demand, as they do not require palladium-containing catalytic converters. The USA has effectively diversified its sources of palladium, exhibiting remarkable resilience in the face of potential supply challenges from key palladium producers. Palladium and copper prices exhibit a moderately positive linear relationship due to their intertwined supply chains and market dynamics. Although the supply chain of nickel is also connected to that of palladium, their prices exhibit a very weak positive correlation.

## Figures and Tables

**Table 1:** Palladium distribution coefficient values during copper and nickel smelting processes from the literature.

Cu Smelting				
Temperature (°C)	*pO*_2_ (atm)	Slag System	Approx. Palladium Distribution Coefficient, *L^Cu/S^*	Reference
1300	10^−10^–10^−7^	FeO_x_–SiO_2_–CaO–MgO^sat.^	10^3^	[38]
1300	10^−9^–10^−8^	FeO_x_–SiO_2_–Al_2_O_3_–CaO	10^4^	[39]
1450	10^−10^–10^−5^	Al_2_O_3_–CaO–SiO_2_–Cu_2_O–MgO^sat.^	10^3^	[40]
1300	10^−9^–10^−5^	FeO_x_–CaO	10^3^	[41]
Ni smelting				
Temperature (°C)	*pO*_2_ (atm)	Slag system	Approx. Palladium Distribution Coefficient, *L^Ni/S^*	Reference
1350–1450	10^−8^–10^−7^	FeO_x_–MgO–SiO_2_	10^4^	[42]

**Table 2:** Pearson correlation coefficient values obtained.

*x_i_*	*y_i_*	Pearson Correlation Coefficient (*r_xy_*)
Yearly average palladium imports from Russia to USA	Yearly average palladium prices	−0.15
Yearly average palladium imports from South Africa to USA		−0.34
Yearly average total palladium imports to USA		−0.61

## Data Availability

The datasets used and/or analyzed during the current study are available from T. Michałek (tomaszm@agh.edu.pl) upon reasonable request.
